# Differential functional dysconnectivity of caudate nucleus subdivisions in Parkinson’s disease

**DOI:** 10.18632/aging.103628

**Published:** 2020-08-31

**Authors:** Xiuqin Jia, Zhenyu Pan, Huimin Chen, Zhijiang Wang, Kun Li, Xuemei Wang, Zhan Wang, Huizi Ma, Tao Feng, Qi Yang

**Affiliations:** 1 Department of Radiology, Beijing Chaoyang Hospital, Capital Medical University, Beijing, China; 2 Department of Movement Disorders, Center for Neurology, Beijing Tiantan Hospital, Capital Medical University, Beijing, China; 3 China National Clinical Research Center for Neurological Diseases, Beijing, China; 4 Parkinson's Disease Center, Beijing Institute for Brain Disorders, Capital Medical University, Beijing, China; 5 Peking University Sixth Hospital (Institute of Mental Health), Beijing, China; 6 NHC Key Laboratory of Mental Health (Peking University), Beijing, China; 7 National Clinical Research Centerfor Mental Disorders (Peking University Sixth Hospital), Beijing, China; 8 Equal contribution

**Keywords:** Parkinson's disease, caudate nucleus subdivision, dopamine, functional connectivity, fMRI

## Abstract

Caudate dopaminergic dysfunction is implied in the pathophysiology of patients with Parkinson’s disease (PD). Still, connectivity specificities of the caudate nucleus (CN) subdivisions and the effect of dopamine are poorly understood. We collected MRI and neuropsychological data from 34 PD patients and 26 age- and sex-matched healthy elderly individuals (HEs) in this study. Resting-state functional connectivity analysis revealed that compared to the other CN subdivisions, the CN head was more strongly connected to the default mode network (DMN), the CN body to the frontoparietal network (FPN), and the CN tail to the visual network in HEs. PD patients off medication showed reduced connectivity within all these subdivision networks. In PD patients on medication, functional connectivity in the CN head network was significantly improved in the medial prefrontal cortex and in the body network it was improved in the dorsolateral prefrontal cortex. These improvements contributed to ameliorated motivation and cognitive function in PD patients. Our results highlighted the specific alterations and dopamine modulation in these CN subdivision networks in PD, which may provide insight into the pathophysiology and therapeutics of this disease.

## INTRODUCTION

Caudate dopaminergic dysfunction underlies the pathophysiology of patients with Parkinson’s disease (PD) [1–6]. The nigrostriatal dopamine deficiency in PD can be relieved by treatment with levodopa (L-dopa). However, the connectivity specificities of the caudate nucleus (CN) subdivisions and the effect of dopamine remain unexplored.

The caudate works in concert with the cortical areas to support different behaviors [7]. A previous study in PD patients that treated the caudate as a functionally uniform region has demonstrated a loss of cortico-caudate coupling in the early stage of this disease during sequence learning [8]. However, the caudate subdivisions topographically participate in differential cortico-striatal circuits [9]. More recent studies have divided the CN into the head, body, and tail [10–13], or dorsal and ventral regions based on the boundaries of their anatomical features [14–16], which are largely overlay the CN head and body, respectively.

Evidence has demonstrated that the ventral CN (head) is connected to the ventral/medial prefrontal cortex, whereas the dorsal CN (body) is connected to the dorsal/lateral prefrontal cortex, both structurally and functionally [11, 12, 15, 17, 18]. The tail of CN sometimes combines with the CN body and gets relatively less attention. Studies of the CN tail have revealed that it is associated with visual saccade and encodes long-term memory of object values [19, 20]. Studies have pointed out that the uniformity of the caudate may cover abnormalities of caudate subdivisions in patients with PD.

Resting-state functional connectivity (rs-FC) provides a sensitive measure of synchronous neural activity. The aim of this study was to highlight specific CN subdivision networks in healthy elderly individuals (HEs) and to explore the connectivity disruption and the role of dopamine in these specific networks in patients with PD. We hypothesized that caudate dysfunction in PD might affect the connectivity of CN subdivisions in different ways, and these abnormalities might be modulated by dopamine treatment.

## RESULTS

### Demographic results

As shown in Table 1, significantly lower MoCA (*p* < 0.001) and higher HAMD scores (*p* < 0.001) were detected in patients with PD compared to HEs. No significant difference was found in age and sex between HEs and patients with PD.

**Table 1:** Demographic and clinical characteristics.

	**HE (*n* = 26)**	**PD (*n* = 34)**	***p*-value**
Age (years)	56.12 ± 10.07	60.41 ± 11.14	0.128
Sex (female/male)	16/10	16/18	0.265
Disease duration (years)	—	2.27 ± 2.19	—
Hoehn and Yahr stage	—	1.59 ± 0.50	—
L-DOPA dose (mg/day)	—	134.19 ± 41.09	—
UPDRS part-I	—	2.61 ± 1.91	—
UPDRS part-II	—	8.09 ± 3.41	—
UPDRS part-IV	—	3.38 ± 1.18	—
UPDRS part-III ON	—	16.76 ± 6.76	—
UPDRS part-III OFF	—	21.24 ± 8.26	—
MoCA^#^	27.58 ± 1.53	23.53 ± 3.63	<0.001
HAMD-24^#^	0.38 ± 0.85	5.59 ± 6.79	<0.001

Note: Data are expressed as mean ± standard deviation. Sex data were analyzed with χ^2^ test. Other *p* values were derived from the independent two-sample *t*-test except for ^*#*^ that was derived from the Mann-Whitney test. Abbreviations: UPDRS, unified Parkinson's disease rating scale; MoCA, Montreal Cognitive Assessment; HAMD-24, Hamilton depression rating scale with 24 items.

### VBM results of GM

Whole brain VBM analysis found that no significant GM atrophy was detected in PD patients compared to HEs. Specifically, the GM volumes in the subdivisions of CN were further extracted and compared between the two groups. It was found that no significant difference was detected between HE and PD groups in the CN head (464.34 ± 53.19 for HE vs. 463.79 ± 56.09, *p* = 0.97), body (412.75 ± 54.73 for HE vs. 413.38 ± 48.57 for PD, *p* = 0.96), and tail (39.15 ± 4.84 for HE vs. 39.77 ± 5.58 for PD, *p* = 0.628), respectively.

### Specific subdivision networks of the CN in the HE

**Figure 1:**
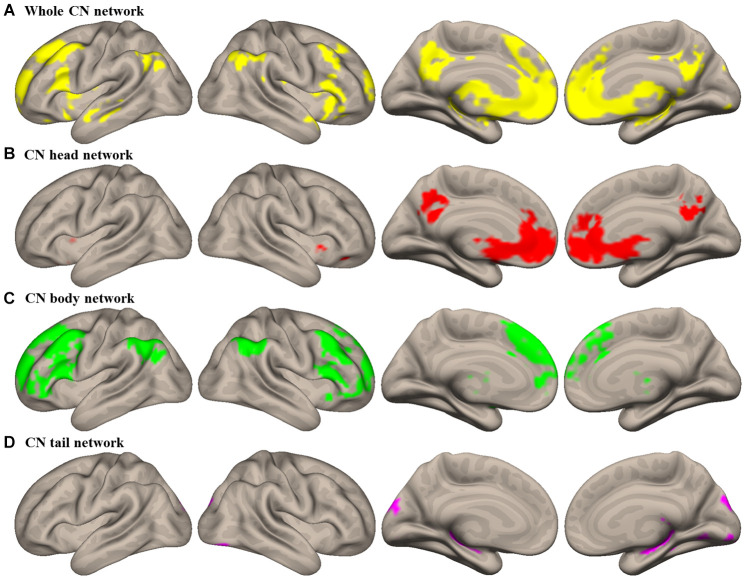
**Intrinsic connectivity networks of caudate nucleus (CN) subdivisions in healthy elderly (HE).** (**A**) Whole CN network; (**B**) CN head network; (**C**) CN body network; (**D**) CN tail network. Results were illustrated at an uncorrected voxel-wise height threshold of *p* < 0.001 combined with an FWE-corrected cluster-wise threshold of *p* < 0.001

**Figure 2:**
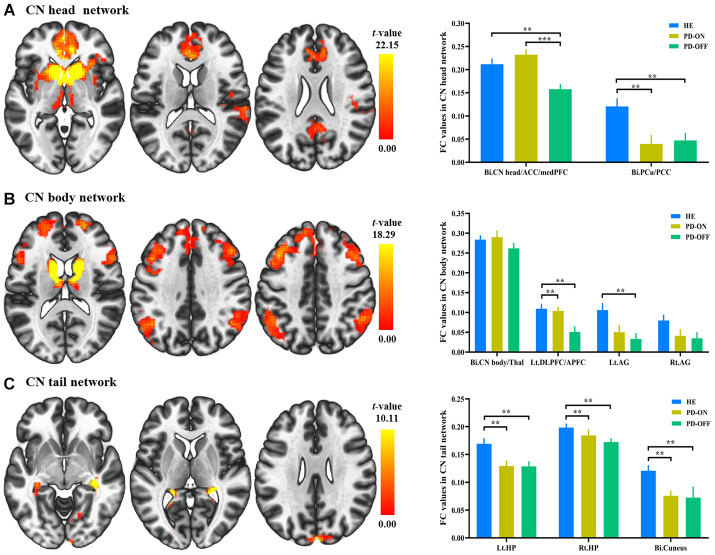
**Functional connectivity (FC) changes in caudate nucleus (CN) subdivisions between PD and healthy elderly (HE), and between PD OFF-medication and ON-medication.** (**A**) CN head network; (**B**) CN body network; (**C**) CN tail network. Results were illustrated at an uncorrected voxel-wise height threshold of *p* < 0.001 combined with an FWE-corrected cluster-wise threshold of *p* < 0.001. Bar graphs demonstrated FC values in the subdivision networks in HE and patients with PD ON-medication and OFF-medication; ^**^, *p* < 0.005; ^***^, *p* < 0.001. Abbreviations: FC, functional connectivity; PD-OFF, PD OFF-medication; PD-ON, PD ON-medication.

CN subdivision networks in HEs were identified in the whole CN network (Figure 1A and Supplementary Table 1). Compared to the other CN subdivisions, the head showed a specific higher connectivity with the anterior cingulate cortex (ACC) extending into medial prefrontal cortex (MPFC) and the CN head, and the precuneus (PCu) extending into the posterior cingulate cortex (PCC) bilaterally (see Table 2 and Figure 1B). The body showed specific higher connectivity with the CN body extending into the thalamus, the dorsolateral prefrontal cortex (DLPFC) extending into the anterior prefrontal cortex (APFC), and the angular gyrus (AG), bilaterally (see Table 2 and Figure 2C). The tail showed higher connectivity with the hippocampus and occipital cortex bilaterally (see Table 2 and Figure 1D). Altered FC in the CN subdivision networks were revealed in PD OFF-mediation and ON-medication (see Supplementary Figure 1).

**Table 2:** Connectivity networks specific to the caudate nucleus subdivisions in healthy elderly.

**Anatomical regions**	**Cluster size (voxel)**	**MNI (x, y, z)**	***T*-value**
**CN head network**			
Bi. CN head/ACC/MPFC	2030	(9, 15, 0)	22.15
Bi. PCu/PCC	278	(-9, -42, 39)	4.91
**CN body network**			
Bi. CN body/Thal	831	(12, 6, 15)	18.29
Lt. DLPFC/APFC	2081	(-48, 21, 21)	6.32
Lt. AG	209	(-45, -57, 39)	5.28
Rt. AG	238	(51, -51, 42)	5.03
**CN tail network**			
Lt. Hippocampus	79	(-24, -39, 6)	10.11
Rt. Hippocampus	148	(18, -24, 18)	13.17
Bi. Cuneus	237	(3, -93, 27)	5.12

Note: Results were thresholded based on an uncorrected voxel-wise height threshold of *p* < 0.001 combined with an FWE-corrected cluster-wise threshold of *p* < 0.001. Abbreviations: CN, caudate nucleus; ACC, anterior cingulate cortex; MPFC, medial prefrontal cortex; PCu, precuenus; PCC, posterior cingulate cortex; Thal, thalamus; DLPFC, dorsolateral prefrontal cortex; APFC, anterior prefrontal cortex; AG, angular gyrus; Bi, bilateral; Lt, left; Rt, right.

### Altered functional connectivity and dopamine modulation in CN subdivision networks in PD

These regions in the specific CN subdivision network were defined as regions of interest (ROIs) for the confirmation analyses. All FC values in each ROI were extracted and analyzed. Patients with PD OFF-medication exhibited a significantly decreased connectivity within the CN head network compared to HEs (*t* = -3.217, *p* = 0.002 for the ACC/MPFC/CN head cluster and *t* = -3.016, *p* = 0.004 for the PCu/PCC cluster). When L-dopa was administered to patients with PD, a significantly improved connectivity was revealed in the CN head/ACC/MPFC cluster and this improvement returned to the normal level (ON-medication vs. OFF-medication, *t* = 5.605, *p* < 0.001; ON-medication vs. HE, *t* = 1.217, *p* = 0.228) (see Figure 2A).

Within the CN body network, significantly decreased connectivity was revealed in the DLPFC/APFC cluster (*t* = -3.080, *p* = 0.003) and the left AG (*t* = -3.157, *p* = 0.002) in PD patients OFF-medication compared to HEs. A significant improvement in the DLPFC/APFC cluster was found after administration of L-dopa (ON-medication vs. OFF-medication, *t* = 2.97, *p* = 0.006; ON-medication vs. HE, *t* = -0.343, *p* = 0.733) (see Figure 2B).

Within the CN tail network, the reduced connectivity in the bilateral hippocampus (*t* = -2.983, *p* = 0.004 for the left; *t* = -2.576, *p* = 0.012 for the right) and bilateral cuneus (*t* = -3.340, *p* = 0.001) was exhibited in PD OFF-medication compared to HE (see Figure 2C). No significant changes were identified in the CN tail network in patients with PD when administered L-dopa.

### Exploratory analysis of dopamine effect in PD

**Figure 3:**
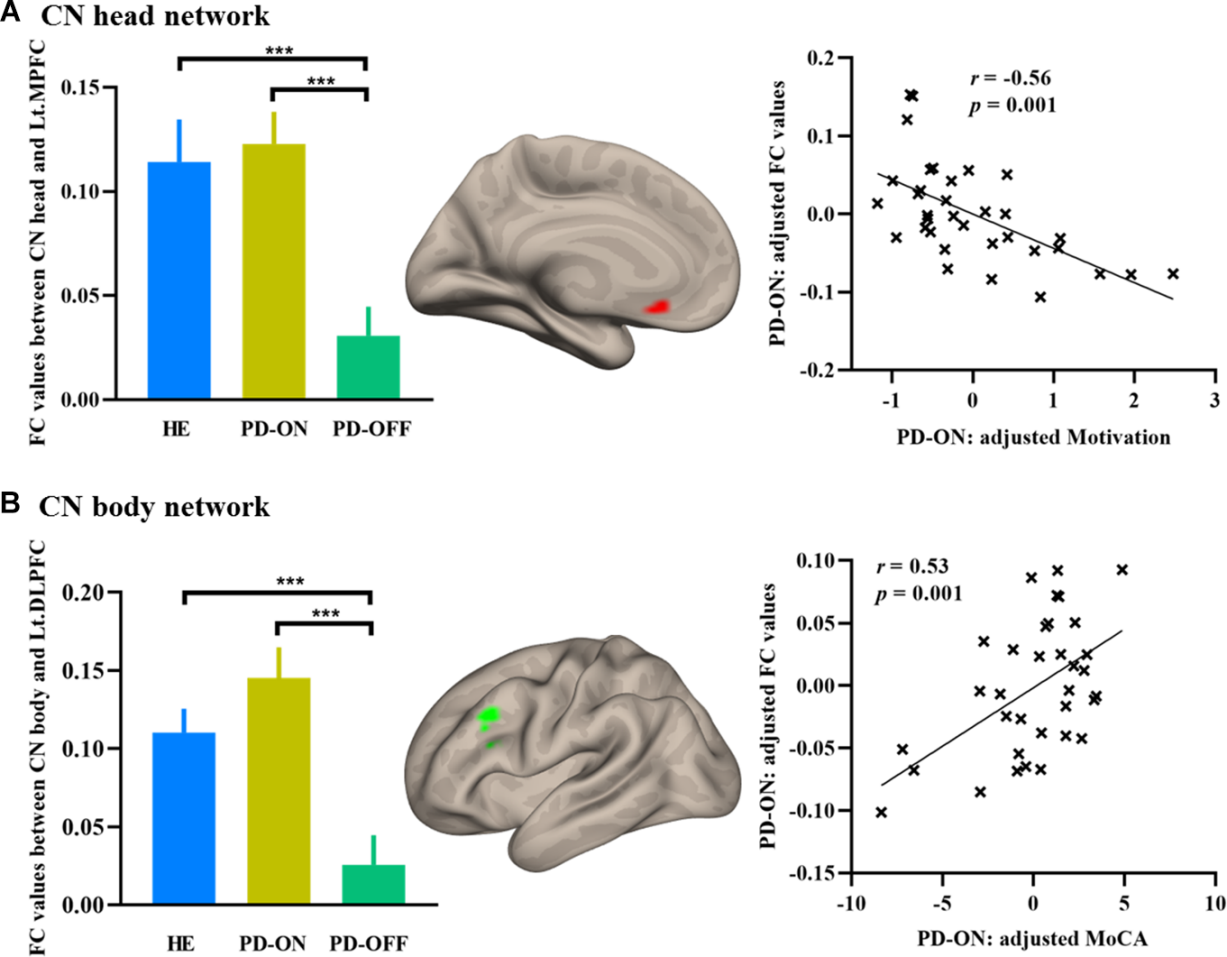
**Increased FC in PD ON-medication.** (**A**) Increased FC in the MPFC in CN head network; (**B**) Increased FC in the DLPFC in CN body network. Bar graphs showed the extracted FC values in HE and PD. Scatterplots showed the relationship between FC values and neuropsychological measurements. Abbreviations: FC, functional connectivity; MPFC, medial prefrontal cortex; DLPFC, dorsolateral prefrontal cortex; MoCA, Montreal Cognitive Assessment; HE, healthy elderly; PD-OFF, PD OFF-medication; PD-ON, PD ON-medication. ^***^, *p* < 0.001.

Three voxel-wise exploratory analyses were conducted within each subdivision network to refine the regions affected most by the dopamine administration within each CN subdivision network. The left MPFC (MNI coordinate: *x* = -9, *y* = 21, *z* = -9; *t* = 3.46) in the CN head network was significantly modulated by dopamine treatment in PD. The mean FC strength was significantly negatively correlated with the motivation measured by UPDRS part I in patients with PD (see Figure 3A). The left DLFPC (MNI coordinate: *x* = -45, *y* = 21, *z* = 36; *t* = 4.53) in the CN body network was significantly modulated by dopamine treatment in PD. Mean functional connectivity strength was significantly positively correlated with MoCA score in patients with PD (see Figure 3B). No voxels showed significant dopamine effect in the CN tail network.

## DISCUSSION

The present study explored the neural pathomechanisms of caudate dopaminergic deficiency in PD. Our data provide evidence for dissociable functional connectivity networks associated with the CN subdivisions in HEs [11–15]. Disrupted functional connectivity was identified in CN subdivisions in PD patients in concert with dopaminergic deficiency. When dopamine was administered, increased functional connectivity between the CN head and DMN was associated with ameliorated motivation. In contrast, the enhanced functional connectivity between the CN body and the FPN correlated with improved cognitive function in PD. These findings provide a deeper insight into the neural mechanisms underlying the pathophysiology and therapeutics of this disease.

The current findings showed that specific connectivity between the CN head and the DMN supports self-referential processing [27]. Converging evidence has shown that this network of the CN head in the DMN is associated with a greater ability for the cognitive control of impulsive behaviors [11], and motivational and automatic processes [28]. The decreased CN head functional connectivity in the DMN in patients with PD is consistent with the non-motor characteristics of PD and psychiatric disturbances caused by caudate dopaminergic pathophysiology [29]. After dopamine administration, the improved functional connectivity between the CN head and the MPFC/ACC provides direct evidence of specific damage. The increased connectivity after dopamine administration between the CN head and the MPFC (hub region of DMN) restored a normal level and contributed to the enhanced motivation measured with the UPDRS part I (item 4) in patients with PD. This finding suggests a self-amelioration of disrupted motivation after dopamine supply [16].

In keeping with earlier studies [11, 12], the CN body was strongly connected to regions in the FPN, including the DLPFC, AG, and the bilateral cerebellum. These results are supported by previous diffusion tensor imaging (DTI) tractography showing that the CN body (analogous to “dorsal caudate”) is linked with the DLPFC [18]. Repeated transcranial magnetic stimulation (rTMS) of the DLPFC modulates dopamine release specifically in the dorsal caudate nucleus [30]. Our current findings in the CN body network showed that functional connectivity strength between the CN body and the frontoparietal cortex was disrupted in concert with dopamine depletion. In patients with PD, an increase in functional connectivity between the CN body and the DLPFC was restored to the average level after dopamine administration. This compensated for dopaminergic loss with increased neuronal firing and, consequently, increased intrinsic functional connectivity. This increased connection strength was positively associated with the cognitive function measured with MoCA and implicates the DLPFC-caudate body circuit in the neural pathomechanisms underlying the cognitive decline in this disease.

The function of the CN tail has received less attention. However, there have been several intriguing findings that imply the tail plays a vital role in visual processing, in particular visual memory/learning processes [19, 20, 31–33]. We found that the CN tail was strongly connected to the hippocampus and occipital cortex bilaterally in HEs. Compared to HEs, PD patients showed significantly less bilateral hippocampal connection in the CN tail network. No significant changes were found in the CN tail network after dopamine treatment in PD patients.

Only PD patients with early-stage disease were included in the present study. This was an effort to eliminate heterogeneity and reduce the effect of long-term dopaminergic medication on brain networks. Several studies have shown that L-dopa modulates caudate activity in PD, even at moderate and advanced stages [34, 35]. This requires further investigation if the observed effect of L-dopa during early stages of PD continues to demonstrate a protective effect at later stages.

Previous studies have reported loss of caudate volume in patients with PD, but lack consistency. For example, caudate atrophy was previously reported in early drug-naive PD patients [1]. By contrast, Hattori et al. failed to detect any differences between PD patients and healthy controls [36]. These inconsistences might be due to heterogeneity among patients, including differences in disease stage and medications. In line with previous studies [36, 37], we detected no volumetric differences in the CN subdivision between PD patients and HEs. The caudate volume loss may not be specific to PD patients, but may be part of normal aging [1]. Nonetheless, this caudate volume loss may worsen in advanced PD due to the severe striatal dopaminergic deficiency.

The present study characterized the abnormalities in the functional connectivity within each caudate subdivision network that occurs with striatal dopamine depletion in PD. The amelioration in these connection networks after dopamine administration highlights that the neural substrates for the pathophysiological changes associates with this disease. These findings show that PD patients have dysfunctional caudate coupling affecting the caudate head, body, and tail in different ways. They also shed light on the pathophysiological mechanisms underlying PD and provide insight into the therapeutics of the non-motor symptoms in this disease. The lack of comprehensive neuropsychological assessments currently hampers our ability to examine the associations between the CN subdivision networks and specific cognitive domains. This will be addressed in a future study.

## MATERIALS AND METHODS

### Participants

In this study, 34 early stage PD patients (18 males, 57.11 ± 11.85 years old vs. 16 females, 64.13 ± 9.27 years old) with Hoehn and Yahr (H&Y) stage of I or II, meeting the UK Bank criteria for the diagnosis of PD [21], were recruited. All PD participants were examined in their ON*-*medication and OFF*-*medication states (at least after a 12-hour withdrawal of anti-Parkinson medication). Patients received L-dopa 30 minutes after receiving 10 mg domperidone on an empty stomach in the morning. L-dopa dosage was determined by the regular effective dosage of the patient. ON-medication examinations were administered an hour later when L-dopa reaches its peak plasma dose. The HE comprised 26 volunteers (10 males, 56.12 ± 10.07 years old), who were matched for age and sex ratio. This study was approved by the Research Ethics Committee of Beijing Tiantan Hospital, Capital Medical University. Written informed consent was obtained from each participant.

### Neuropsychological assessments

The clinical characteristics were assessed ON-medication except UPDRS part-III which was evaluated both ON- and OFF-medication. The disease stage was scored using the H&Y stage score and disease severity was captured by the UPDRS part-III. Assessments of the neuropsychological state included the Montreal Cognitive Assessment (MoCA) and the Hamilton Depression Rating Scale (HAMD-24) [22].

### MRI data acquisition

MRI data were acquired using a Siemens 3 Tesla Prisma MRI system (Siemens, Erlangen, Germany) by using a 32-channel head coil. Foam padding and headphones were used to limit head motion and reduce scanner noise. Participants were instructed to keep still and remain motionless. High-resolution structural images were acquired using 3D T1-weighted magnetization-prepared rapid gradient echo (MPRAGE). Scan parameters were as follows: TR = 2300 ms, TE = 2.3 ms, flip angle = 8°, matrix size =256 × 256, 192 1 mm sagittal slices. Resting-state functional MR imaging (rs-fMRI) data were obtained by using an echo-planar imaging sequence that lasted 8 minutes 20 seconds (200 volumes) with the parameters: TR = 2500 ms, TE = 30 ms, flip angle = 90°, FOV = 200 mm × 200 mm, matrix size =70 × 70, 43 slices, and slice thickness = 3 mm.

### Voxel-based morphometry (VBM) analysis

To determine the structural abnormalities in patients, we performed a VBM analysis for all anatomical images of PD and HE using SPM12 (http://www.fil.ion.ucl.ac.uk/spm) and CAT12 (http://www.neuro.uni-jena.de/cat/). T1-weighted images were segmented using the unified segmentation model into gray matter (GM), white matter (WM), and cerebrospinal fluid (CSF) based on tissue probability maps (TPMs) in MNI space. The spatially normalized GM maps were modulated by the Jacobian determinant of the deformation field and corrected for individual brain sizes. The modulated, normalized GM images (voxel size 1.5 × 1.5 × 1.5 mm^3^) were smoothed with an 8-mm full width at half maximum isotropic Gaussian kernel.

### Rs-functional connectivity analysis

**Figure 4:**
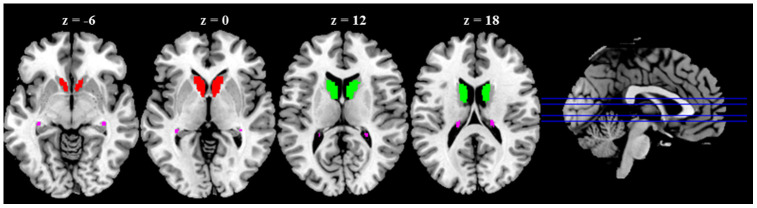
**Anatomical seeds of caudate nucleus (CN) subdivisions.** Red color represents CN head; Green color represents CN body; Violet color represents CN tail.

Rs-fMRI data were preprocessed using SPM12. Seed-to-voxel correlation analysis was carried out by Data Processing and Analysis for (Resting-State) Brain Imaging (DPABI) V4.3 [23]. The first ten functional images were discarded to minimize the fluctuation of the MRI signal in the initial stage of scanning. The remaining 190 images of each subject were first corrected for slice timing to reduce the within-scan acquisition time differences between slices and then realigned to eliminate the influence of head motion during the experiment. All subjects included in the present study exhibited head motion less than 1.5 mm in any of the x, y, or z directions and less than 1.5^°^ of any angular dimensions. Next, the realigned images were co-registered to T1 images, spatially normalized into MNI space using transformations from segmentation, and resampled voxel size into 3 × 3 × 3 mm^3^. Subsequently, the functional images were smoothed with a 4-mm FWHM isotropic Gaussian kernel. After preprocessing, images were then band-pass filtered to 0.008 ~ 0.09 Hz to reduce noise. Further denoising steps included regression of six motion parameters and their first-order derivatives, regression of WM and CSF signals following the CompCor strategy [24] and a linear detrending. The seed-regions of the bilateral caudate head, body, and tail were defined based on WFU_PickAtlas [25] (Figure 4). The correlation coefficients between the seed voxels and all other brain voxels were computed to generate correlation maps. For group analyses, the correlation coefficients were converted to *z*-value using Fisher's *r*-to-*z* transformation [26].

### Identifying the CN subdivision networks

The three individual connectivity maps (obtained from the CN head, body, and tail) of each HE were entered into a factorial model in SPM12 with age and sex as nuisance variates. The whole CN network was first identified (defined as the regions connected with any of the three CN subdivisions with *F-*contrast in SPM). Functional connectivity maps specific to each seed region were obtained by comparing to the other two CN subdivisions.

### Confirmatory analysis of changes in CN subdivision network and dopamine effect in PD

These regions within each specific CN subdivision network were defined as regions of interest (ROIs) for the confirmatory analyses. Two sample *t*-tests were performed to explore the dysconnectivity in PD compared to HE. Paired *t*-test was conducted to examine the dopamine modulation within each subdivision network in PD ON-medication compared to OFF-medication.

### Exploratory analysis for dopamine modulation in the CN subdivision networks

An exploratory analysis was performed additionally to ascertain which voxels were affected by the dopamine administration within each CN subdivision network. Partial correlation analyses were examined between the mean functional connectivity values in these voxels and clinical performances controlled for nuisance variables.

### Statistical analysis

Two-sample *t*-test was performed for the demographic, clinical measurements, and extracted CN subdivision volume between PD patients and HE using SPSS 22. GM volume and functional connectivity results were reported based on an uncorrected voxel-wise height threshold of *p* < 0.001 combined with an FWE-corrected cluster-wise threshold of *p* < 0.001. Brain regions were localized with xjView (http://www.alivelearn.net/xjview). Correlations between these alterations and neuropsychological performances were examined. Results for confirmatory ROIs analysis and correlations were corrected for multiple comparisons using the Bonferroni correction (*p* < 0.05).

## Supplementary Material

undefined

undefined
