# Supplementary material for: Differential functional dysconnectivity of caudate nucleus subdivisions in Parkinson’s disease
Source: Aging (Albany NY). 2020 Aug 31;12(16):16183–94. doi: 10.18632/aging.103628 (PMC7485745; doi:10.18632/aging.103628)
Supplement: undefined [file aging-12-103628-s002..pdf]

## SUPPLEMENTARY FIGURE

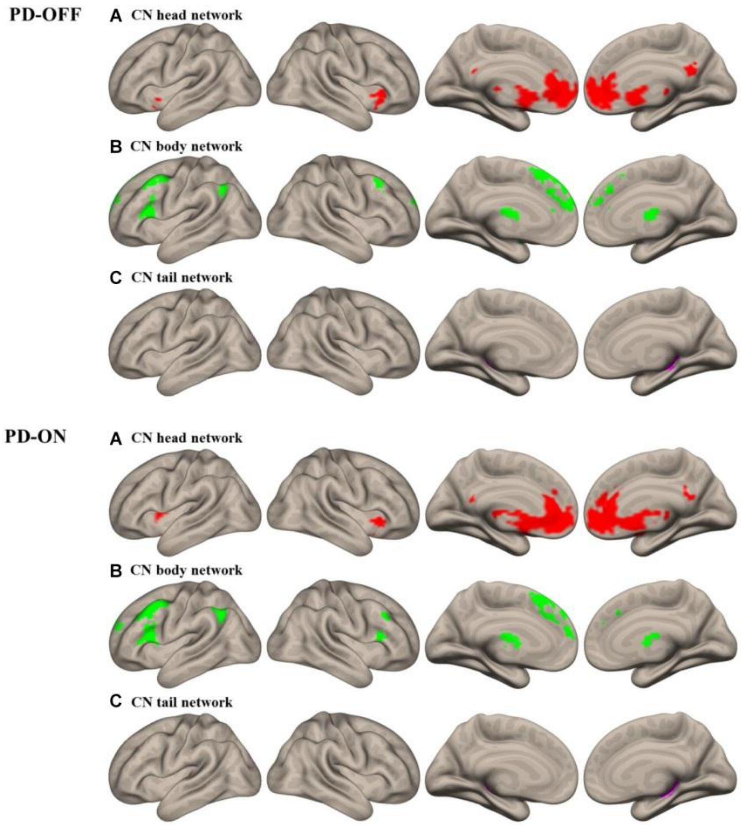


**Supplementary Figure 1. Intrinsic connectivity networks of caudate nucleus (CN) subdivisions in PD.** (**A**) CN head network; (**B**) CN body network; (**C**) CN tail network. Results were illustrated at uncorrected voxel-wise height threshold of $p < 0.001$ combined with an FWE-corrected cluster-wise threshold of $p < 0.001$. Abbreviations: PD-OFF, PD OFF-medication; PD-ON, PD ON-medication.
