# Supplementary material for: Differential functional dysconnectivity of caudate nucleus subdivisions in Parkinson’s disease
Source: Aging (Albany NY). 2020 Aug 31;12(16):16183–94. doi: 10.18632/aging.103628 (PMC7485745; doi:10.18632/aging.103628)
Supplement: undefined [file aging-12-103628-s001..pdf]

SUPPLEMENTARY TABLE

Supplementary Table 1. Intrinsic connectivity networks of the whole caudate nucleus.

Anatomical regions	Cluster size (voxel)	MNI (x, y, z)	*F*-value
**HE**			
Bi.MPFC/ACC/Frontal_Mid/CN/Thal	6732	(9, 15, 0)	217.28
Bi.PCu/PCC	662	(-6, -42, 39)	14.80
Lt.AG	352	(-48, -78, 33)	10.38
Rt.AG	390	(54, -63, 36)	12.30
Lt.Temporal_Mid	165	(-66, -24, -9)	10.36
**PD ON-medication**			
Bi.MPFC/ACC/Frontal_Mid/CN/Thal	6882	(9, 12, 0)	336.00
Lt.AG	180	(-54, -51, 39)	9.02
Lt.Temporal_Sup	146	(-54, -18, 6)	15.31
Rt.Temporal_Sup	136	(60, 0, -3)	13.55
**PD OFF-medication**			
Bi.MPFC/ACC/Frontal_Mid/CN/Thal	6034	(12, 6, 12)	312.26
Lt.Precental	276	(-39, 6, 51)	14.81
Lt.AG	112	(-54, -57, 36)	11.93
Rt.Temporal_Sup	169	(-51, -30, 12)	10.33

Note: Results were reported at an uncorrected voxel-wise height threshold of p < 0.001 combined with an FWE-corrected cluster-wise threshold of p < 0001. Abbreviations:
CN, caudate nucleus; ACC, anterior cingulate cortex; MPFC, medial prefrontal cortex; PCu, precuenus; PCC, posterior cingulate cortex; Thal, thalamus; AG, angular gyrus; Mid, middle; Sup, superior; Bi, bilateral; Lt, left; Rt, right; HE, healthy elderly; PD, Parkinson’s disease.
